# CRISPR/Cas9 mutagenesis of the *Arabidopsis* GROWTH-REGULATING FACTOR (GRF) gene family

**DOI:** 10.3389/fgeed.2023.1251557

**Published:** 2023-10-16

**Authors:** Juan Angulo, Christopher P. Astin, Olivia Bauer, Kelan J. Blash, Natalee M. Bowen, Nneoma J. Chukwudinma, Austin S. DiNofrio, Donald O. Faletti, Alexa M. Ghulam, Chloe M. Gusinde-Duffy, Kamaria J. Horace, Andrew M. Ingram, Kylie E. Isaack, Geon Jeong, Randolph J. Kiser, Jason S. Kobylanski, Madeline R. Long, Grace A. Manning, Julie M. Morales, Kevin H. Nguyen, Robin T. Pham, Monthip H. Phillips, Tanner W. Reel, Jenny E. Seo, Hiep D. Vo, Alexander M. Wukoson, Kathryn A. Yeary, Grace Y. Zheng, Wolfgang Lukowitz

**Affiliations:** ^1^ Department of Plant Biology, University of Georgia, Athens, GA, United States; ^2^ Division of Biology, University of Georgia, Athens, GA, United States

**Keywords:** T-DNA vector, transgene-free, seed bio-fluorescence selection, LED illumination, teaching lab, BsaI, sgRNA multiplexing, tRNA spacer

## Abstract

Genome editing in plants typically relies on T-DNA plasmids that are mobilized by *Agrobacterium*-mediated transformation to deliver the CRISPR/Cas machinery. Here, we introduce a series of CRISPR/Cas9 T-DNA vectors for minimal settings, such as teaching labs. Gene-specific targeting sequences can be inserted as annealed short oligonucleotides in a single straightforward cloning step. Fluorescent markers expressed in mature seeds enable reliable selection of transgenic or transgene-free individuals using a combination of inexpensive LED lamps and colored-glass alternative filters. Testing these tools on the *Arabidopsis* GROWTH-REGULATING FACTOR (GRF) genes, we were able to create a collection of predicted null mutations in all nine family members with little effort. We then explored the effects of simultaneously targeting two, four and eight GRF genes on the rate of induced mutations at each target locus. In our hands, multiplexing was associated with pronounced disparities: while mutation rates at some loci remained consistently high, mutation rates at other loci dropped dramatically with increasing number of single guide RNA species, thereby preventing a systematic mutagenesis of the family.

## 1 Introduction

GROWTH-REGULATING FACTORs (GRFs) form a small family of DNA-binding proteins found in land plants and their algal precursors (reviewed in Omidbakhshfard et al., 2015; Kim, 2019; Liebsch and Palatnik, 2020). GRF activity affects proliferative growth in many organs (including shoot and root apical meristems, leaf primordia, flowers, seeds) and in many plant species, suggesting that this family plays a central role in regulating cell division rates according to positional cues or endogenous and environmental signals. Consistent with this view, genetic variation in GRF genes has been reported to underpin quantitative traits relevant to agriculture. A prominent example is a dominant, naturally occurring allele of rice GRF4 associated with increased grain size (Che et al., 2015; Duan et al., 2015; Hu et al., 2015; Li et al., 2016; Sun et al., 2016); this mutation maps to the binding site of micro-RNA miR396, an evolutionary conserved negative regulator of nearly all GRF genes, enabling mutant transcripts to escape miR396-mediated degradation and accumulate to higher amounts. More recently, forced expression of GRF genes was found to promote organogenesis in cultured tissue of plant species that are difficult to regenerate by conventional methods (reviewed in Lee and Wang, 2023). The *Arabidopsis* genome encodes nine GRF proteins representing five ancient phylogenetic clades. However, a critical assessment of the specific contributions these different GRF clades make to growth and development has been hampered by pervasive genetic redundancy (Kim et al., 2003; Horiguchi et al., 2005; Hewezi et al., 2012; Kim et al., 2012; Lee et al., 2012) as well as the lack of *bona fide* null alleles for several family members.

Genome editing with CRISPR/Cas9 nucleases offers an approach for circumventing both of these limitations. CRISPR/Cas complexes can be programmed to bind virtually any DNA sequence, enabling applications as diverse as chemical modification of target DNA, directed manipulation of gene transcription, or genome editing by homologous recombination (reviewed in Knott and Doudna, 2019; Soyars et al., 2018; Manghwar et al., 2019; Zhang et al., 2019). Natural CRISPR/Cas systems predominantly function as sequence-specific endo-nucleases directed against invasive DNA (Terns and Terns, 2011), and a first technical application was to re-purpose this activity for inducing mutations at specific loci in a genome of interest. Simplified variants of the *Streptococcus pyogenes* system, consisting of the Cas9 apoprotein in complex with a single guide RNA (sgRNA; Jinek et al., 2019), are now widely used for site-directed mutagenesis. Multiple loci can be targeted simultaneously by co-expressing appropriate sgRNAs, and various platforms enabling multiplexed CRISPR-based screens in plants have been developed with this aim in mind (reviewed in Najera et al., 2019; Hassan et al., 2021; Gaillochet et al., 2021). Although perhaps not yet widely employed, successful multiplex-based mutagenesis experiments have been reported. Ma et al. (2015) simultaneously mutagenized eight rice FT-like genes and identified primary transgenic plants that carried mutations in seven of them; these plants showed visible phenotypes consistent with a loss of FT-like function. Xie et al. (2015) targeted five rice MAP kinase genes and found that ∼50% of the primary transgenics harbored editing events in all targets. Zhang et al. (2016) recovered a plant that harbored mutations in all of the six *Arabidopsis* PYR-like genes that were targeted from a sample of only 15 primary transgenics; the mutations were germline-transmitted and enabled rapid assembly of a six-fold mutant. Suttmann et al. (2021) simultaneously expressed 24 sgRNAs in *Arabidopsis* and succeeded in identifying a 12-fold mutant plant by a screening for a combination of visible and molecular phenotypes.

We were interested in including CRISPR/Cas9-based mutagenesis in a lab course on plant molecular biology and therefore needed to simplify the workflow as much as possible. An attractive platform was provided by a series of T-DNA vectors designed for adding targeting sequences (the ∼20 nucleotide segments at the 5′end of sgRNAs homologous to the target loci) as short, synthetic oligonucleotide-assemblies in a single cloning step (Xing et al., 2014; Wang et al., 2015). We modified these vectors by introducing marker genes enabling selection and counter-selection of transgenic plants on the basis of seed fluorescence (similar to Gao et al., 2016). In addition, we verified that seed fluorescence could be detected with standard dissecting microscopes using an inexpensive external illumination consisting of high-intensity LED lights and colored-glass alternative emission filters. Utilizing these tools as part of a teaching lab, we first mutagenized all nine *Arabidopsis* GRF genes individually and established a collection of reference alleles with frameshift mutations prior to the conserved DNA-binding domain. We then explored approaches to mutagenize to the entire family as means of directly probing gene function. When targeting two, four and eight GRF genes simultaneously, we found that multiplexing was associated with widely disparate mutation rates at different loci: with increasing number of sgRNA species, mutation rates at some targets dropped drastically while remaining consistently high at others. This effect ultimately prevented an even mutagenesis of the GRF family.

## 2 Materials and methods

### 2.1 Seed stocks, plant growth and transformation

The Columbia accession of *Arabidopsis thaliana* served as a wild type strain for transformation and CRISPR/Cas9 mutagenesis. Plants were grown under constant fluorescent light at ∼25°C on commercial potting mix (RediEarth, Sun Gro Horticulture) with slow-release fertilizer (Osmocote). For germination in sterile culture, seeds were surface sterilized in 70% ethanol for 1 min, rinsed twice in 96% ethanol, briefly air dried, and transferred to plates containing 1% sucrose, 1% agar (Sigma A-1296) and 0.5x MS salts (MP Biomedicals 2623022). The GV3101 strain of *Agrobacterium* was used for plant transformation following the floral dip protocol (Clough and Brent, 1998). Transgenic plant experiments were carried out in accordance with relevant biosafety regulations and guidelines. We verified by PCR that the seed stocks of GRF reference alleles deposited with the *Arabidopsis* Stock Center, Ohio, are free of CRISPR/Cas9 T-DNAs.

### 2.2 Imaging

Seed fluorescence was imaged with an Olympus SZX12 stereo-microscope equipped with a Moticam 3.0 plus digital camera and a Kramer Scientific Quad internal illumination module connected to an X-cite 120 mercury lamp; filter sets for GFP (Kramer Scientific 184, with narrow band emission) and propidium iodide (Kramer Scientific 816) were used for YFP- and Tomato-fluorescence, respectively. The instrument did not have appropriate filters for imaging CFP-fluorescence. The improvised LED lamps we assessed as a low-cost alternative illumination are documented in Supplementary Presentation S1.

### 2.3 Plasmid construction

T-DNAs expressing a single or two sgRNA were generated by conventional cloning as described by Xing et al. (2014). Briefly, T-DNA vectors were linearized by restriction with *BsaI* (we found that incubation overnight at 45°C gave best results) and gel purified. For single-sgRNA constructs, two non-phosphorylated complementary oligonucleotides encoding the 20 nt gene-specific targeting sequences plus appropriate overlapping ends for cloning (Supplementary Data Sheet S1) were mixed (50 µM each, 2X SSC buffer) and annealed in a temperature gradient (96°C–20°C); 1 µL of the annealing reaction was combined with ∼100 ng vector fragment (with intact 5′-phosphate ends) and ligated with T4 ligase (1 h, room temperature).

For constructs with two sgRNAs, fragments containing the gene-specific targeting sequences, the sgRNA backbone and either a U6-29 promoter or a tRNA spacer were produced by four-primer PCR as described by Xing et al. (2014) using a proofreading enzyme (Q5 polymerase, New England Biolabs, M0491). Briefly, the reactions contained a pair of inner and outer primers at a ratio of 1:20 (50 nM and 1 μM, respectively); inner primers were designed to anneal to template plasmids and in addition encoded the targeting sequence of the sgRNAs; outer primers were designed to use the DNA fragments produced by the inner primers during the first few PCR cycles as template and in addition contained sequences required for generating vector-compatible ends by *BsaI* digestion; in this way, the length of all primers could be limited to ∼40 nt (Supplementary Data Sheet S1). Two plasmids were used as templates: pGEM-2t, derived form a custom synthetic fragment encoding a sgRNA backbone as well as an alanine tRNA spacer (see Supplementary Data Sheet S1 for an annotated sequence listing; Addgene 159752; ABRC CD3-2856); and pCBC-DT1DT2, containing a sgRNA backbone as well as a U6-29 promoter (Xing et al., 2014; Addgene 50590). Ligations included ∼50 ng of the PCR product (*BsaI*-digested; gel-purified) and ∼100 ng vector fragment (*BsaI*-digested; terminal phosphates removed by treatment with shrimp alkaline phosphatase, ThermoFisher 78390; gel-purified) and were incubated at 16°C overnight.

T-DNAs expressing four sgRNAs were assembled from a *BsaI*-linearized vector fragment and three PCR fragments using an NEBuilder kit (E2621, New England Biolabs). Appropriate PCR fragments were generated in three- or four-primer reactions analogous to the ones described above, with the outside primer containing the 23–25 nt overlaps required for the assembly reaction (Supplementary Data Sheet S1). The sgRNA genes of all constructs were verified by Sanger sequencing.

### 2.4 Detection and sequencing of mutant alleles

Gene-specific targeting sequences were selected such that CRISPR/Cas9 would cause a double strand break within the recognition sequence of a restriction enzyme, enabling the detection of induced mutations with PCR-based markers (Supplementary Data Sheet S1). The same PCR protocols were used to amplify germline-transmitted mutant alleles for Sanger sequencing with either internal or PCR primers (Supplementary Data Sheet S1).

### 2.5 Amplicon sequencing and data analysis

For the purpose of estimating the mutation frequencies at different GRF loci in plants expressing four or eight sgRNA species, two complementary DNA samples were generated: the “4-targets” sample was extracted from 1151 T2 seedlings: 527 mutagenized with the “1,256” construct (230 from 5 wild type parents, 297 from 5 *grf9-6* parents), 582 T2 mutagenized with the “3,478” construct (297 from 5 wild type, 285 from 5 *grf-6* parents), and 42 controls (described below); the “8-targets” sample was extracted from 1310 T3 seedlings: 1,265 mutagenized with both constructs (form 20 families of *grf9-6*/+ parents), and 51 controls. 10 independent transformation events of each construct are represented in these populations (see Results for pedigree). From each T1 parent or T2 family, ∼50 non fluorescent seeds were selected and grown on plate for 7 days; germinated seedlings were tallied and then combined to generate the two samples; the sample material was ground in liquid nitrogen and the DNA extracted following a modified CTAB protocol (Murray and Thompson, 1980).

For each GRF gene, ∼200 bp amplicons that included the CRISPR/Cas9 target sites were generated; the PCR primers contained tails for library construction ((Supplementary Data Sheet S1). Amplicons were barcoded and sequenced on an Illumina platform at the UGA Georgia Genomics and Bioinformatics Core (dna.uga.edu). The resulting reads were aligned to a 60 nt wild type reference sequence centered around the predicted CRISPR/Cas9 cut site and analyzed for insertion and/or deletion events using AGEseq (Xue and Tsai, 2015).

GRF9 was not mutagenized in the experiment, such that the amplicon could be used to assess representation. 51% of the seedlings represented in “4-targets” were from *grf9-6* parents (582/1,151), and 51% of the mapped GRF9 amplicon reads contained the *grf9-6* mutation; similarly, 97% of the seedlings represented in “8-targets” were *grf6-1/+* (1,265/1,310), and 50% of all mapped reads contained the *grf-6* mutation. In addition, the samples were spiked with 24, 12, and 6 seedlings from *grf9-3/grf9-4*, *grf9-1/grf9-2*, and *grf9-7/grf9-8* parents, respectively. The *grf9-2* allele is a ∼180 bp deletion-insertion event that could not be amplified with our primers; *grf9-3*, *grf9-4*, *grf9-7*, and *grf9-8* harbor small deletions flanking the CRISPR/Cas9 cut site (indicated by a star): ttg*------atg, gtg-*------atg, gtg-*ccg, tgg*-cgt, respectively; *grf9-1* contains a 9 bp insertion (upper case letters): tgg*AGTTTCGGAgga. The representation of these alleles in the two DNA samples is summarized in Supplementary Table S1.

AGEseq reported 243 distinct insertion-deletion events in the sequences of GRF1-GRF8 amplicons (for summary statistics see Supplementary Table S2). We discarded 30 of these events as likely artifacts, since they were supported by fewer than 50% of the reads expected to be generated from a single allele present in one seedling of the sample (the representation of the *grf9* spike-in controls had a standard deviation of ∼25%, suggesting that this cut-off is inclusive).

## 3 Results

### 3.1 CRISPR/Cas9 T-DNA vectors for selecting and counter-selecting transgenic seeds on the basis of bio-fluorescence

**FIGURE 1:**
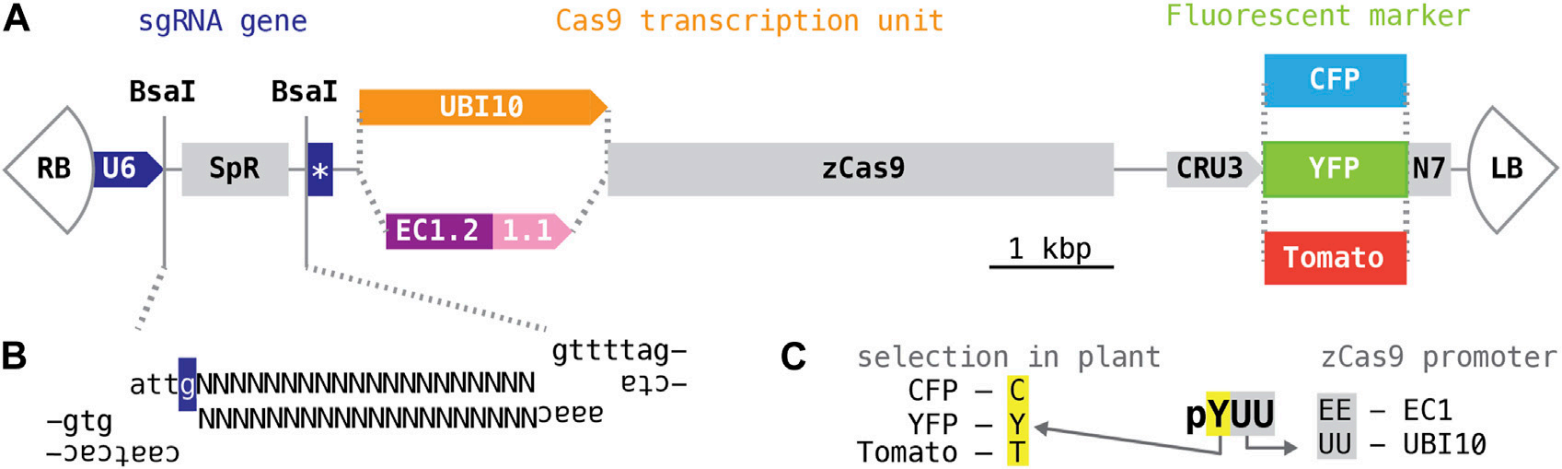
CRISPR/Cas9 T-DNAs for selection and counter-selection on the basis of seed fluorescence. **(A)** Schematic organization of T-DNA vectors. All plasmids are variants of the vectors described Xing, Wang and colleagues (2014 and 2015). “RB” and “LB”, right and left T-DNA border; “U6”, U6-26 promoter; “SpR”, buffer sequence; star, sgRNA scaffold and U6 terminator; “UBI10”, polyubiquitin10 promoter; “EC1.2 1.1”, egg cell1.1 promoter with egg cell1.2 enhancer; “zCas9”, Cas9 coding sequence optimized for maize; “CRU3”, cruciferin 3 promoter; “YFP”, “CFP”, “Tomato”, yellow, cyan, red fluorescent protein coding sequence; “N7”, nuclear localization signal of At4g19150 (Cutler et al., 2000). **(B)** Annealed oligonucleotides encoding a single gene-specific targeting sequence (represented with “Ns”) or more complex assemblies for expression of multiple sgRNAs for the same T-DNA can be inserted into the *BsaI* cloning site; the ends generated by *BsaI* have different 5′overhangs; the “g” highlighted in dark blue represents the transcriptional start site of the U6-26 promoter. **(C)** Vectors are named after the fluorescent marker (first letter) and the promoter driving Cas9 (remaining two letters).

T-DNAs of the Cambia family (derived from pPZP; Hajdukiewicz et al., 1994) are among the most widely used plasmid vectors for plant transformation. Their relatively small size and high copy numbers in *E. coli* simplify molecular cloning; their pVP1 origin ensures effective propagation in *Agrobacterium* and high transformation rates with a wide range of plant species. Building on pHEE401, a Cambia T-DNA adapted for genome editing with CRISPR/Cas9 nucleases (Wang et al., 2015), we created a series of vectors for selection and counter-selection of transgenics on the basis of seed fluorescence. Toward this end, the hygromycin resistance marker of pHEE401E was replaced with fluorescent protein variants of different colors expressed from the promoter of the *Arabidopsis* seed storage albumin A1 gene (CRU3, At4g28520) (Figure 1A), specifically: a cyan fluorescent protein (CFP; Cubitt et al., 1999), a “Venus” yellow fluorescent protein (YFP; Nagai et al., 2002), both modified from *Aequorea*, and a monomeric “Tomato” red fluorescent protein (Shaner et al., 2004), modified from *Discosoma*; all fluorescent protein variants were fused to an N-terminal nuclear localization signal (N7; Cutler et al., 2000).

The CRISPR/Cas9 module of pHEE401E contains two genes, one producing the sgRNA and one producing the Cas9 mRNA. The sgRNA transcription unit consists of the *Arabidopsis* U6-26 (At3g13855) polymerase III-dependent promoter, followed by a buffer segment (SpR), a 75 bp sequence encoding the sgRNA scaffold, and the *Arabidopsis* U6-26 terminator. The buffer segment is designed to be removed by digestion with *BsaI*, a restriction enzyme cutting outside of its recognition sequence (GGTCTCN_1/5_). *BsaI* digestion leaves incompatible 5′-overhangs precisely at the transcriptional start site of the sgRNA gene, enabling insertion of a synthetic 19–20 base pair targeting sequence specific for gene of interest in a single, technically straightforward cloning step; larger fragments for expression of multiple sgRNA species may be inserted in a similar manner (Xing et al., 2014; Wang et al., 2015) (Figure 1B). No changes were made to this segment of the T-DNA.

The Cas9 transcription unit consists of egg cell-specific promoter and enhancer elements taken from the *Arabidopsis* EC1.1 and EC1.2 genes (At1g76750 and At2g21740) followed by an open reading frame optimized for maize codon usage (zCas9). Ideally, egg cell-specific activity of zCas9 would induce mutations very early in embryonic development, generating heterozygous or bi-allelic mutant primary transgenics rather than mosaics (Wang et al., 2015). In addition, we also employed a promoter taken from the *Arabidopsis* polyubiquitin10 gene (At4g05320) (Figure 1A); this promoter drives robust transcription in a broad range of cell types and is commonly used for genome editing (Ma et al., 2016; Soyars et al., 2018). Annotated sequence listings of the resulting six CRISPR/Cas9 T-DNA vectors can be found in Supplementary Data Sheet S2; plasmid DNA is available through repositories.

### 3.2 Low-cost LED illumination for detecting fluorescence in mature seeds

**FIGURE 2:**
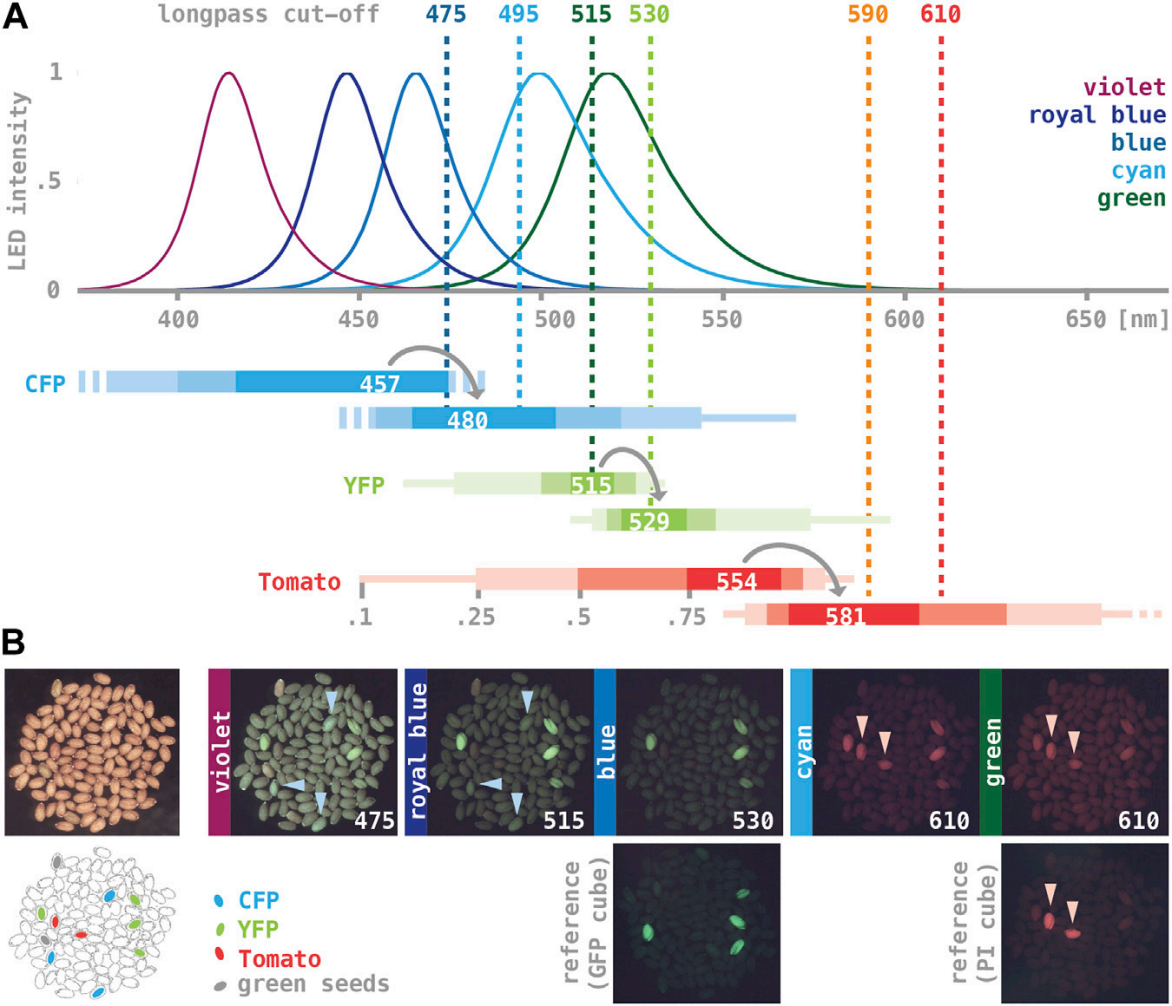
LED/coated glass filter illumination for imaging seed fluorescence. **(A)** Dotted lines mark the cut-off of longpass colored-glass alternative filters (Newport 20CGA-475, -495, −515, −530, −590, −610; values from www.newport.com). Curves show normalized emission spectra of five high-intensity LED assemblies (“violet”: Luxeon Star SZ-05-U9, “royal-blue”: H4; “blue”: H3; “cyan”: H2; “green”: H1; “lime”: H9; graphs adapted from www.luxeonstar.com). Horizontal bars represent the excitation (top) and emission spectra (bottom) of three fluorescent proteins below (maxima are listed; shaded intervals mark 0.75, 0.5, 0.25, and 0.1 of the respective maxima; values from www.fpbase.com; Lambert, 2019). **(B)** A sample of control seeds imaged with different illuminations. A bright field image and a trace of the seeds are shown on the left, with the position of CFP-, YFP-, and Tomato-expressing transgenics, as well as two greenish, chlorophyll-containing seeds highlighted. The LED assemblies and the cut-off of colored-glass alternative filters used to generate the images are noted on each panel. Images taken with the benchmark illumination are shown in the bottom row. Light-blue arrowheads in “475” and “515” point to CFP-expressing seeds, pink arrowheads in “610” and the PI benchmark point to Tomato-expressing seeds. All fluorescence images were taken with the same camera settings except for exposure time, which was either 0.5 s (515, 530, GFP benchmark) or 1 s (others).

Bio-fluorescence markers are valued for their ease of use, but commercial fluorescence illuminations can be prohibitively expensive for settings such as teaching labs. Motivated by a note in *The Worm Breeder’s Gazette* (Chin-Sang and Zhong, 2008), we explored the performance of high-intensity LED lights combined with colored-glass alternative emission filters as a means of providing external epi-fluorescence illumination for standard dissecting microscopes. We tried six LED assemblies producing relatively narrow spectra of light with maxima ranging between 415 and 540 nm and six longpass emission filters with cut-off values ranging between 475 and 610 nm (Figure 2A). Our test sample was a collection of wild type seeds spiked with a small number of seeds expressing either CFP, YFP, or Tomato from one of the T-DNAs described above (Figure 2B). As a benchmark, we imaged the test sample using an Olympus SZX12 stereo-microscope fitted with an internal fluorescence illumination module (see Materials and Methods; YFP was imaged using a GFP filter cube, Tomato using a propidium iodide filter cube; no appropriate filters were available for CFP).

Seeds expressing YFP could be readily imaged using “royal blue” or “blue” LED lights (∼440 and ∼470 nm maximum) and 515 or 530 nm emission filters (Figure 2B). These illuminations produced a brighter background compared to the benchmark, but the overall contrast remained high. CFP-expressing seeds were best detectable when illuminated with a “violet” LED light (∼410 nm maximum) combined with 475 nm emission filter, and Tomato-expressing seeds when illuminated with “cyan” or “green” LED lights (∼500 and ∼520 nm maximum) combined with 590 or 610 nm excitation filters. However, CFP- and Tomato-fluorescence was significantly dimmer, with a relatively low signal-to-noise ratios (Figure 2B); moreover, YFP-expressing seeds imaged with these combinations often appeared as bright as the CFP- or Tomato-expressing seeds, suggesting significant bleed-through. Remnants of chlorophyll present in some of the seeds caused noticeable red auto-fluorescence, in particular when viewed with “violet” LED lights.

*Arabidopsis* is typically transformed by infiltrating live plants with *Agrobacterium* (Clough and Brent, 1998); in most cases, less than one percent of the seeds harvested from treated plants will be transgenic. As a stringent practical test, we used the benchmark instrument as well as a standard dissecting scope and illumination by LED/colored-glass alternative filters to select primary transgenic events form samples of seeds bulk-harvested after infiltration. For the YFP marker, more than 80% of the fluorescent seeds detected with the benchmark instrument were also detected using LEDs and colored-glass alternative filters. For the Tomato marker, this ratio was much lower (approximately 20%), implying that only transformation events resulting in relatively strong Tomato-fluorescence can be reliably scored.

In summary, “royal-blue” or “blue” LED lights in combination with 515 or 530 nm longpass emission filters provide effective illumination for imaging YFP fluorescence in seeds. CFP and Tomato fluorescence can be detected using “violet” and “cyan” or “green” LED lights combined with a 475 or 610 nm longpass emission filter, respectively–however, sensitivity is comparatively low. A list of components for assembling the described light sources can be found in the Supplementary Presentation S1, and stocks of fluorescent seeds that can be used as reference for imaging are available from the *Arabidopsis* Stock Center, Ohio.

### 3.3 Targeting individual GRF genes: Cas9 expression with the polyubiquitin promoter results in ten-fold higher mutation rates than expression with the EC1 promoter

**FIGURE 3:**
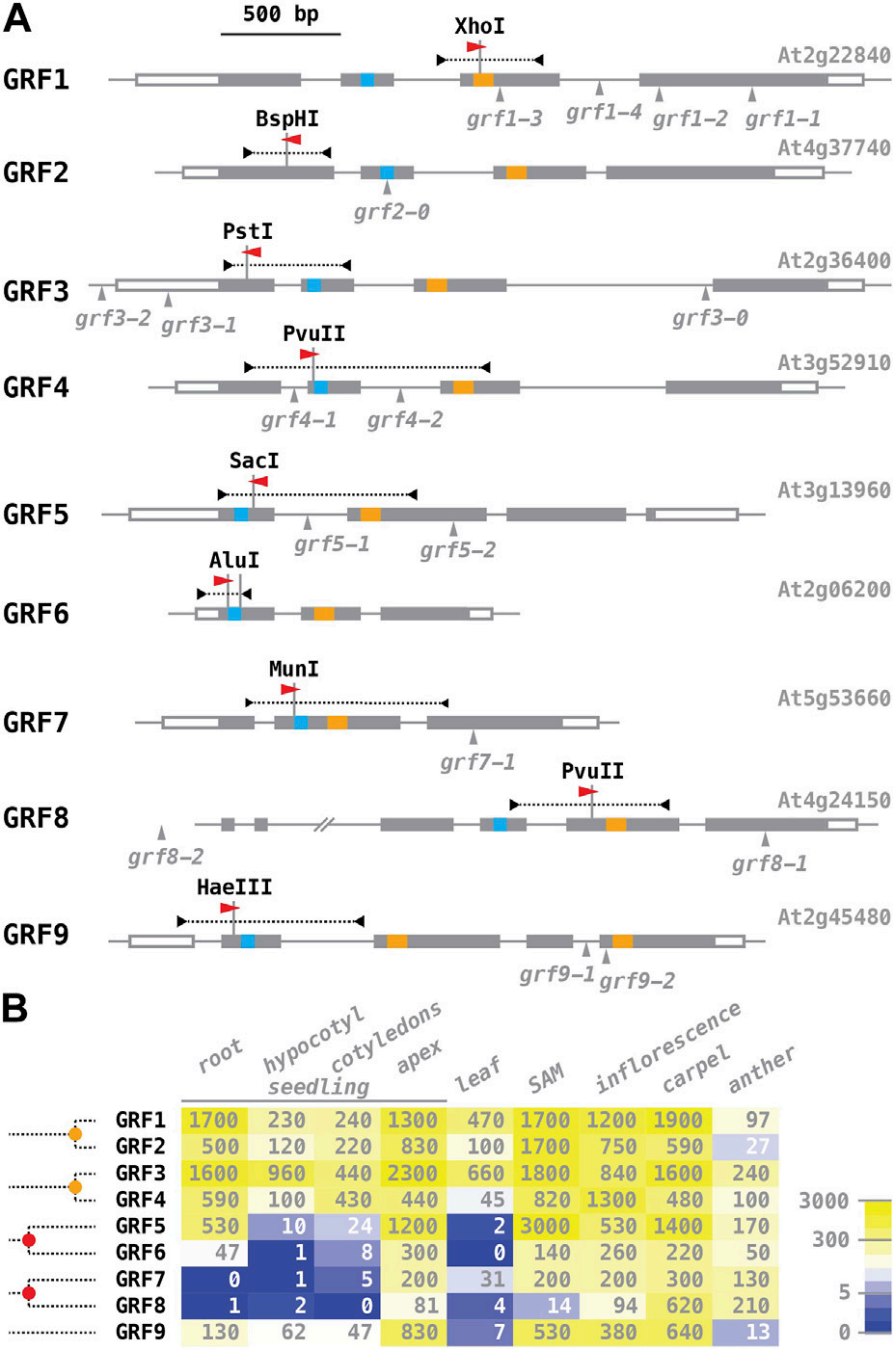
The GROWTH-REGULATING FACTOR (GRF) gene family of *Arabidopsis*. **(A)** Schematic organization of the *Arabidopsis* GRF genes (aligned at the translational start site). Exons are shown as boxes, with coding sequences filled in grey, the QLQ motif in blue, and the WRC motif in orange. The position and direction of protospacer regions, identical to the targeting sequences of sgRNAs, are indicated by a red arrowhead the fragments amplified to screen for induced mutations are shown above as dotted lines, with the restriction sites destroyed by mutations listed. All previously reported GRF alleles are due to T-DNA insertions, mapped below the gene models; they are described in: Kim et al. (2003) - *grf1-1*, *grf1-2*, *grf2-0*, *grf3-0*; Horiguchi et al. (2005) - *grf5-1*, *grf9-1*; Kim and Lee (2006) - *grf4-1*; Hewezi et al. (2012) - *grf1-3*, *grf1-4*, *grf3-1*, *grf3-2*; Kim et al. (2012) - *grf7-1*, *grf8-1*, *grf8-2*; Lee et al. (2018) - *grf5-2*; Omidbakhshfard et al. (2019)—*grf9-2*. **(B)** Overview of GRF mRNA expression (data from travadb.org; Klepikova et al., 2016). Numbers represent the normalized average count per million reads; note that the scale of the color scheme is logarithmic. Samples of “seedlings” were collected 1 day after germination; “apex” represents the shoot meristem and surrounding tissues; “leaf” represents the third rosette leaf at the time of flower opening; “SAM” represents the vegetative shoot apical meristem 8 days after germination; “carpels” and “anthers” were harvested at the time of flower opening. Phylogenetic relationships of the *Arabidopsis* GRF genes are sketched on the right side (after Omidbakhshfard et al., 2015): the five GRF sub-clades found in *Arabidopsis* date back to before the last common ancestor of flowering plants; the alpha whole genome duplication event (∼30–35 million years ago) is marked by an orange dot, the gamma triplication event at the base of the eudicots (∼120 million years ago) by a red dot.

We chose the nine *Arabidopsis* GROWTH-REGULATING FACTORS (GRFs) as a test-case for genome editing with our vectors. GRF proteins appear to be specific to the streptophytes, a phylogenetic group including the land plants and their algal precursors, the charophytes, and are defined by the combination of two structural features: a QLQ domain with the invariant core of QX_3_LX_2_Q, and a WRC domain with a specifically spaced cysteine and histidine residues (Figure 3A; Omidbakhshfard et al., 2015; Kim, 2019; Liebsch and Palatnik, 2020; different spacing of the cystein residues sets the WRD domain apart from C_3_H zinc-fingers; Wang et al., 2008). The WRC domain mediates sequence-specific DNA binding, while the QLQ domain enables protein-protein interactions with co-regulators, most notably the GRF-INTERACTING FACTORs. A phylogenetic analysis reveals that the nine *Arabidopsis* GRF genes represent five ancient clades, dating back to before the last common ancestor of flowering plants (Omidbakhshfard et al., 2015; molecular signatures suggestive of sub-functionalization patterns are discussed in Meng et al., 2022) (Figure 3B). GRF5/GRF6 and GRF7/GRF8 were likely separated in a whole genome triplication event at the base of the eudicots (∼130 million years ago; Jiao et al., 2012); GRF1/GRF2 and GRF3/GRF4 reside on large syntenic blocks (Lee et al., 2012) that were separated in the *α* whole genome duplication (∼30 million years ago, before the split of *Arabidopsis* and *Brassica*; Vision et al., 2000; Ermolaeva et al., 2003). Insertion alleles have been reported for all GRFs except GRF6 (Figure 3A); however, in many cases the insertion sites are in the promoter, in introns, or downstream of the WRC motif, and it is not clear that gene function is completely abolished. According to expression data in the public domain, GRF transcripts appear to accumulate predominantly in tissues with high mitotic rates, such as the shoot apical meristem and reproductive organs (Figure 3B; data compiled from Klepikova et al., 2016; see Lee et al., 2018, for expression of GRF reporter genes in inflorescences).

**FIGURE 4:**
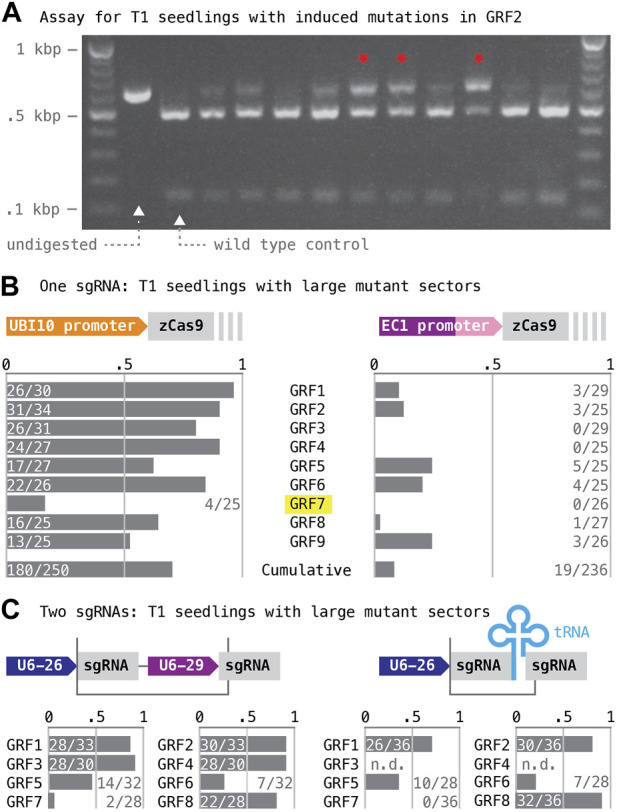
Assessment of mutagenesis targeting GRF genes individually and in pairs. **(A)** The rate of induced mutation in GRF target genes was estimated based on the occurrence of large mutant sectors. Results for 11 T1 seedlings transformed with a construct expressing Cas9 from the EC1 promoter and targeting GRF2 are shown as an example; note that the rate of induced mutations with this construct is low, with most samples showing no or only small apparent sectors; the red stars mark cases where about half or more of the total DNA was undigested–these cases were scored as positive. **(B)** Cas9 apoprotein was expressed either from the polyubiquitin10 (UBQ10, left) or the egg cell-specific EC1 promoter (right). Estimated mutation rates are listed below. **(C)** Different combinations of two sgRNA species were expressed either from separate genes (top left) or from a polycistronic gene with a tRNA spacer (top right); DNA fragments inserted into the *BsaI* cloning site of the vector boxed; “U6-26” and “U6-29”, small RNA promoters. Estimates of the mutation rates at target genes are shown below; targets are listed in the same order as the sgRNAs were arranged on the constructs.

Gene-specific targeting sequences were selected from the CRISPR-Plant database (Xie et al., 2014; www.genome.arizona.edu/crispr2) following two criteria: the sgRNAs had to target an exon upstream of the WRC motif, such that induced lesions would generate frame-shift mutations early in the coding sequence; the predicted Cas9 cut site had to lie within the recognition sequence of a restriction enzyme, such that induced mutations could be detected by PCR and restriction digest (Figure 3B). Annealed oligonucleotides encoding the targeting sequences (Supplementary Data Sheet S1) were inserted into T-DNA vectors expressing Cas9 from either the EC1 or the UBI10 promoter. For each construct, ∼25 primary transgenic (T1) seedlings were selected and tested for mutant sectors; we applied relatively stringent criteria and only counted samples as positive if ∼50% or more of the PCR product remained uncut after restriction digest (Figure 4A; an example with overall low CRISPR/Cas9 activity was chosen to better contrast samples harboring no or small mutant sectors with samples harboring large mutant sectors).

The frequency of seedlings with large mutant sectors served as a proxy for the rate of CRISPR/Cas9-induced mutations at the nine GRF loci (Figure 4B). Our results show that expression of Cas9 with the polyubiquitin10 promoter caused almost ten-fold higher overall mutation rates than expression of Cas9 with the EC1 promoter (UBI10: 0.72, n = 250; EC1: 0.058, n = 236). By comparison, the mutation rates induced by different sgRNAs showed relatively little variability: when Cas9 was expressed from the UBI10 promoter, frequencies of greater than 50% were obtained for all genes except GRF7 (the GRF7 guide also performed poorly when Cas9 was expressed from the EC1 promoter).

### 3.4 A collection of GRF reference alleles

**FIGURE 5:**
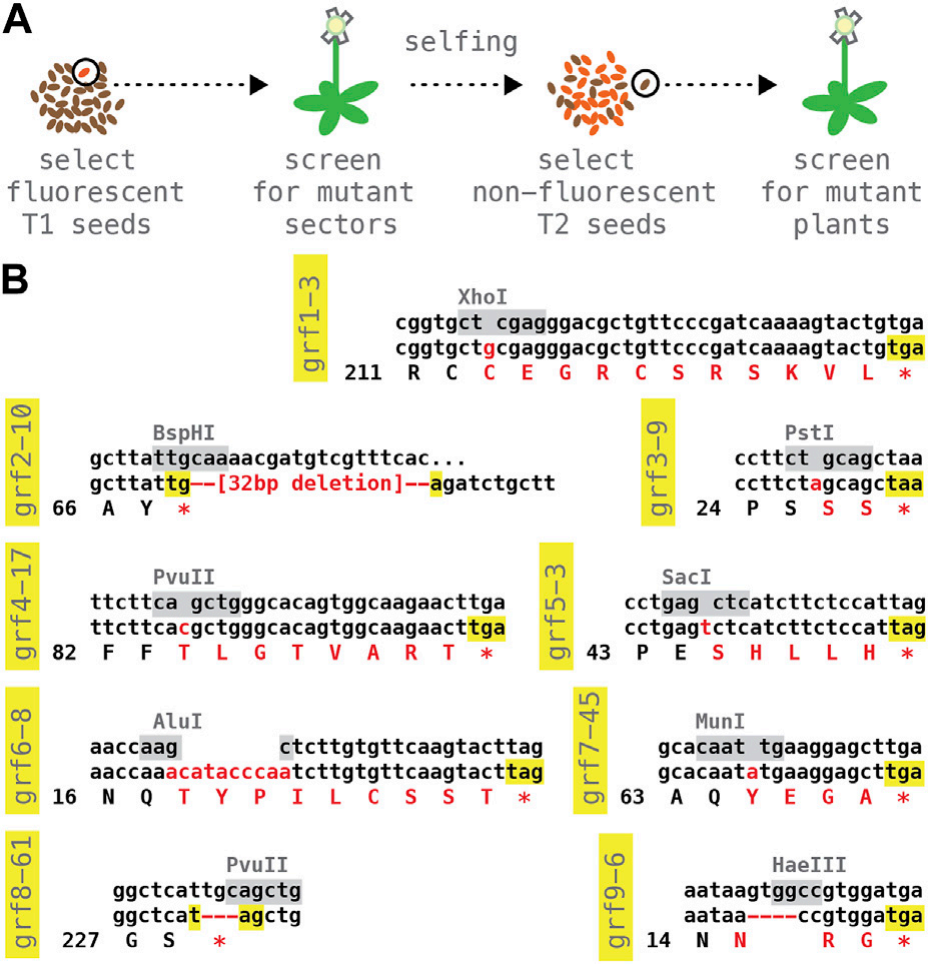
Panel of predicted null alleles in the nine *Arabidopsis* GRF genes. **(A)** Selection and counter-selection scheme for identifying CRISPR/Cas9-induced mutations; see text for details. **(B)** Molecular lesions of reference alleles. The wild type DNA sequence surrounding the CRISPR/Cas9 cut site is listed on top, with restriction site used to identify mutations highlighted in grey; the middle and bottom row show the DNA and predicted protein sequence of the mutant allele; inserted or deleted nucleotides as well as amino acid changes are shown in red. All alleles are predicted to cause a premature stop (highlighted in yellow).

We next isolated germline-transmitted CRISPR/Cas9-induced mutations (Figure 5A). For each target locus, ∼10 T1 plants were grown to maturity and tested for mutant sectors using DNA extracted from rosette leaves or the primary inflorescence; sectored plants were allowed to self-fertilize and their seed harvested; ∼3–6 non-fluorescent, transgene-free T2 seed per positive T1 line were then propagated on soil, and the resulting plants assayed again. Despite the small sample size, mutant alleles were recovered in most cases (GRF1: 3 alleles from 3 T1, testing 3 T2 each; GRF3 and GRF4: 2 alleles from 3 T1, testing 3 T2 each; GRF5 and GRF6: 3 alleles from 7 T1, testing 3 T2 each); only with GRF7 and GRF8 was it necessary to examine the progeny of more than 10 T1 plants.

Stably transmitted GRF alleles were also identified in the non-transgenic progeny of ∼10–20 T1 plants bulk-harvested blindly, without screening for mutant sectors (GRF2: 3 alleles, testing 10 T2 from a pool of 10 T1; GRF8: 1 allele, testing 20 T2 from a pool of 10 T1; GRF9: 5 alleles, testing 20 T2 from a pool of 10 T1). While less well controlled, this simpler selection scheme may reduce time and effort in large-scale experiments.

Sanger sequencing of the induced lesions revealed mostly small insertion/deletion events at the predicted CRISPR/Cas9 cut sites. From this collection, we chose a presumptive null mutation for each of the nine *Arabidopsis* GRF genes (Figure 5B). Homozygous plants could be obtained with all our reference alleles. Neither single mutants nor double-mutants of closely related sister genes (*grf1-3;grf2-10*, *grf3-9;grf4-17*, *grf5-3;grf6-8*, and *grf7-45;grf8-61*) showed obvious abnormalities. Seed samples of single and double mutant lines are have been deposited with the *Arabidopsis* Stock Center, Ohio.

### 3.5 Targeting pairs of GRF genes: similar mutation rates obtained by expressing sgRNAs as independent genes or as part of one polycistronic gene including a tRNA spacer

**FIGURE 6:**
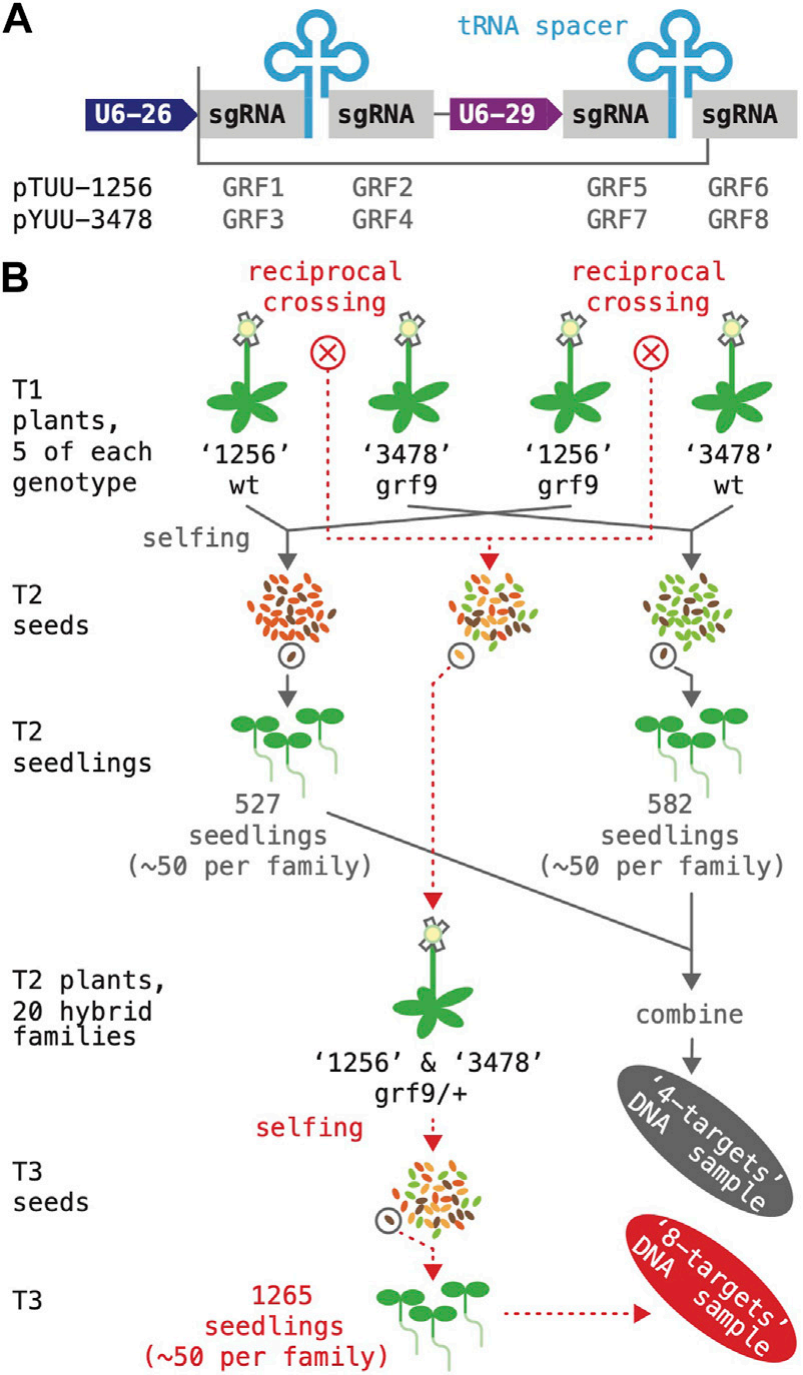
Simultaneous mutagenesis of four and eight GRF genes. **(A)** A schematic of the two T-DNA constructs expressing four sgRNA species are shown on top; DNA fragments inserted into the *BsaI* cloning site of the vector boxed; “U6-26 and U6-29”, small RNA promoters. **(B)** Flow-chart illustrating how the two DNA samples for amplicon sequencing were generated. The “4-targets” sample (grey arrows) represents plants that had been subjected to mutagenesis by either the “1,256” or the “3,478” construct; for each construct, 10 T1 plants were grown to maturity and harvested; ∼50 transgene-free seeds per T1 plant were germinated and combined for DNA extraction. The “8-targets” sample (dotted red lines) represents plants that had been subjected to mutagenesis by both constructs; reciprocal crosses between pairs of “1,256” and “3,478” T1 plants were performed (total of 20 crosses), and T2/F1 plants containing both constructs selected; ∼50 transgene-free seeds per T3/F2 family were germinated and combined for DNA extraction.

An effective simultaneous mutagenesis of multiple loci should significantly reduce the time and effort required to create higher order mutant individuals. Multiple sgRNA species can be expressed in the same plant by placing independent sgRNA genes on a single T-DNA or, alternatively, by constructing a polycistronic transcription unit, in which segments encoding sgRNAs alternate with segments encoding a tRNA; the cellular tRNA processing machinery will excise these tRNA segments post-transcriptionally, liberating the sgRNAs (Xie et al., 2015). We directly compared the efficiency of these two designs by generating T-DNAs targeting pairs of GRF genes (GRF1/2, GRF3/4, GRF5/6, and GRF7/8) either with sgRNAs expressed form separate promoters (U2-26, At3g13855; U6-29, At5g46315) or with sgRNAs derived from a polycistronic transcript (Figure 6). DNA fragments encoding the respective combinations were produced by PCR with primer combinations that included the gene-specific targeting sequences as well as terminal *BsaI* sites (as in Xing et al., 2014; a plasmid containing the U6-29 promoter and a synthetic DNA fragment containing an alanine tRNA sequence served as templates; see Materials and Methods; Supplementary Data Sheet S1). Targeting sequences were the same as before, and Cas9 expression was driven from the UBI10 promoter.

T1 seedlings harboring the constructs were assayed for large mutant sectors as described (Figure 4C; note that due to problems with plant transformation, data for the T-DNA targeting GRF3/4 with sgRNAs separated by a tRNA spacer is missing). Averaged over all target genes, the estimated mutation rates in this experiment were slightly lower than the rates observed previously; but they neither seemed to be strongly affected by how the two sgRNA were generated (Figure 4C; independent promoters: 0.65, n = 246; separated by tRNA: 0.52, n = 200; compared to 0.72, n = 250, when targeted individually) nor by the position of an sgRNA within the construct (5′position–GRF1, GRF5, GRF7: 0.57, n = 82, when targeted individually, compared to 0.41, n = 193, when targeted as part of a pair; 3′position–GRF2, GRF6, GRF8: 0.81, n = 85, when targeted individually, compared to 0.61, n = 118, when targeted as part of a pair). Loci showing the highest mutation rates when targeted individually also tended to show the highest rates when targeted as part of a pair. Interestingly, mutant sectors in the two loci of a pair did not arise independently: the large majority of all T1 seedlings either harbored large mutant sectors at both loci or tested wild type for both (98 and 57, respectively); the remaining seedlings showed mutant sectors only at the locus with the higher overall mutation rate when targeted individually (68, n = 193, combined data for both types of constructs). These findings imply that endonuclease activity in this experiment varied more substantially between different transgenic lines than between CRISPR/Cas9 complexes containing different sgRNA species.

### 3.6 Simultaneous targeting of the GRF family: multiplexing of four or more CRISPR RNAs results in vastly different mutation rates at different target loci

Finally, we explored the effect of further stacking sgRNAs as a means of systematically mutagenizing the entire GRF family. To minimize sequence repetition in the constructs, we combined the two multiplexing approaches assessed before: two small RNA genes driven by a U6-26 and a U6-29 promoter, respectively, were arranged in tandem; each gene produced a poly-cistronic transcript encoding two sgRNAs separated by an alanine tRNA (Figure 6A). Construct “1,256” had a Tomato selectable marker and targeted GRF1, GRF2, GRF5, and GRF6; construct “3,478” had a YFP selectable marker and targeted GRF3, GRF4, GRF7 and GRF8 (see Materials and Methods; Supplementary Data Sheet S1).

We used the two T-DNAs to generate plants in which either four or eight GRF genes were being mutagenized; these plants were also segregating for the *grf9-6* reference allele, since GRF9 was not targeted by either of the T-DNAs (Figure 6B). As a first step, both constructs were transformed separately into wild type as well as *grf-9-6* mutant plants. For each of the four genotypes, five T1 plants harboring a large mutant sector in at least one of the target genes were identified as previously. These plants were allowed to self-fertilize and harvested–yielding 20 families of T2 seeds. In addition, reciprocal crosses were performed between the five wild type plants harboring the “1,256” transgene and the five *grf9-6* plants harboring the “3,478” transgene (total of 10 crosses), as well as the five *grf9-6* plants harboring the “1,256” transgene and the five wild type plants harboring the in the “3,478” transgene (total of 10 crosses). From each cross, ∼5 T2/F1 seed showing Tomato-as well as YFP-fluorescence were propagated on soil; the resulting plants (*grf9-6*/+; hemizygous for both constructs; representing 20 independent transformation events) were allowed to self-pollinate and harvested–yielding 20 families of T3 seeds.

The frequency of CRISPR-Cas9-induced mutations induced was then estimated by amplicon-sequencing. Two DNA samples were prepared for this purpose: the “4-targets” sample represented a population in which four genes were targeted simultaneously (grey in Figure 6B; 527 and 582 seedlings produced by T1 plants harboring “1,256’ and “3,478”, respectively; ∼50 seedlings per family; half of the T1 parents were *grf9-6*); the “8-targets” sample represented a population in which eight genes were targeted simultaneously (red in Figure 6B; 1,265 seedlings total, ∼50 per family; all T2 parents were *grf9-6*/+). Selection of non-fluorescent seeds ensured that, barring sampling errors and artifacts, all sequenced mutations had been germline-transmitted. A small number of seedlings with known mutations in GRF9 was added to both samples for control purposes (see Materials and Methods).

DNA fragments covering the eight predicted CRISPR/Cas9 cut sites were generated by PCR, barcoded, and sequenced on an Illumina platform (details and summary statistics in Materials and Methods; Supplementary Tables S1, S2). We used the AGEseq tool (Analysis of Genome Editing by sequencing; Xue and Tsai, 2015) to determine the frequency of small deletions or insertions in the DNA samples (Supplementary Figure S1). Nearly all lesions mapped exactly to the predicted CRISPR/Cas9 cut site, with the few remaining cases one or two positions off (grey dots in Supplementary Figure S1). The most common types of lesions were single base pair insertions, and deletions of one or two base pairs. Five relatively long insertions, ranging from 15 to 55 bp, were also represented in the collection; two of them originated from the mutagenized GRF locus, the other three contained sequences we were unable to track. Although different target genes showed slightly different spectra of lesions, we saw no evidence for microhomology-based repair at the CRISPR/Cas9 cut site (Sfeir and Symington, 2015; Vu et al., 2017).

**FIGURE 7:**
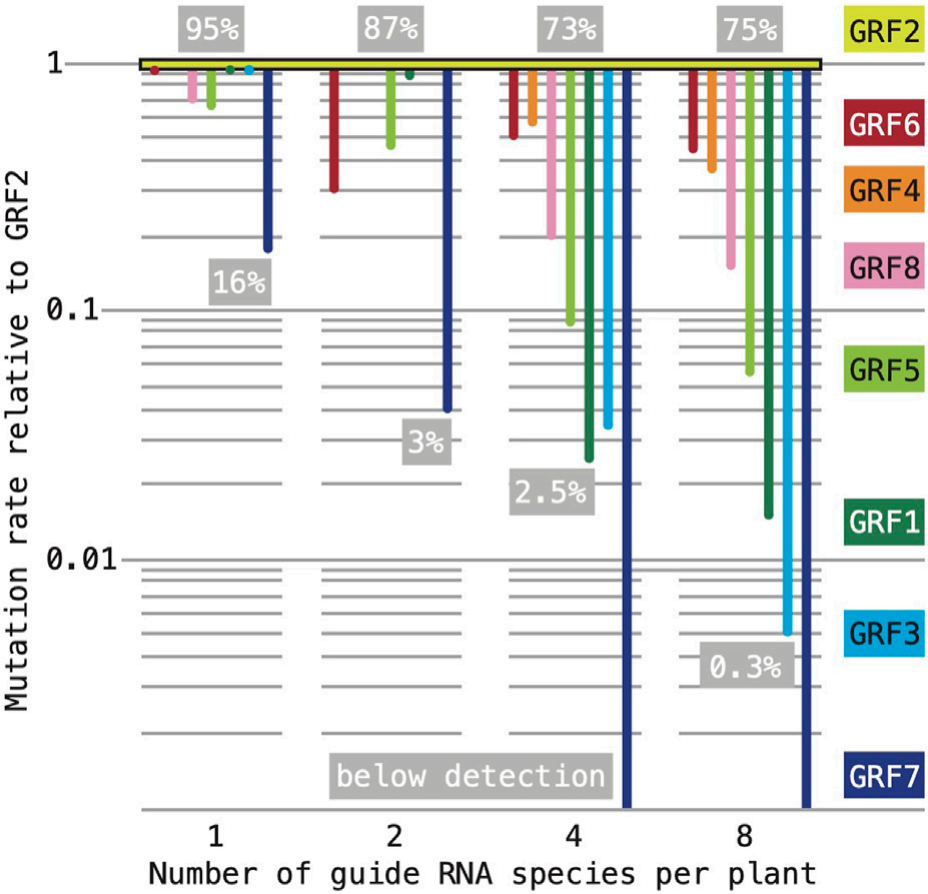
Effect of sgRNA multiplexing on mutation rates at different target loci. Our estimates of mutation rates are not directly comparable: in experiments with one or two sgRNAs, we assessed the frequency of large mutant sectors in ∼24 T1 seedlings; in experiments with four or eight sgRNAs, we assessed the frequency of germline-transmitted mutant alleles in ∼1,000 seedlings. Throughout, the most effective sgRNA was the one targeting GRF2. For the purpose of comparison, the mutation rates estimated in each experiment were normalized to GRF2 (set to “1” on the *y*-axis; note that the scale is logarithmic); the number of sgRNA species in the experiment is listed on the *x*-axis. To convey the range of mutation frequencies within each experiment, the highest and lowest estimates are listed in the grey boxes (expressed as percentage of the total sample).

Overall, the effects of the two constructs varied little between our two populations: the target genes of “1,256” construct were mutated with an average frequency of ∼32% when the construct was acting by itself and ∼29% when acting in combination with “3,478”; for the “3,478”construct, the corresponding values were ∼7% and ∼10%. This finding implies an additive interaction. The perhaps most striking result of the analysis was that mutation frequencies at individual target loci were vastly different and appeared to be strongly affected by multiplexing. A compilation of the estimated mutation rates in all our experiments (normalized to the rates at GRF2, which were consistently highest) reveals that our multiplexing scheme drastically reduced the frequency of lesions at some target genes, while showing only small effects at others (Figure 7). When acting individually, all sgRNAs (except for the guide targeting GRF7) produced large mutant sectors in greater than 50% of the T1 seedlings. The GRF2 sgRNA continued to induce mutations at high rates: 95% individually; 87% in combination with a GRF1 sgRNA; 73% and 75% when combined with three or seven other sgRNA species, respectively. In contrast, the mutation rates associated with the majority of sgRNAs show a more or less steep downward trajectory with increasing number of co-expressed sgRNA species. Thus, our approach did not produce an even mutagenesis of the family.

## 4 Discussion

A plethora of binary vectors is available for genome editing in plants (for an overview see Hassan et al., 2021). Here, we add variants enabling selection and counter-selection of transgenes on the basis of seed fluorescence to the toolbox of CRISPR/Cas9 plasmids created by Xing et al. (2014), Wang et al. (2015). The vectors we describe should be particularly well-suited to small-scale or low-budget projects carried out in minimal settings, including teaching labs: T-DNAs expressing a single sgRNA are generated by ligating a vector fragment (which can be prepared in advance and stocked) with a synthetic oligonucleotide assembly–as rapid, simple, and cheap a cloning procedure as we know; the value of fluorescence-based markers for removing CRISPR/Cas9 transgenes once no longer needed has been recognized before (Gao et al., 2016; Wu et al., 2018; Aliaga-Franco et al., 2019), and we show that fluorescent seeds can be readily identified using a combination of inexpensive LED lights and colored-glass alternative filters. Applying these tools to the *Arabidopsis* GRF gene family, we were able to produce a set of reference alleles harboring frame-shift mutations that truncate the predicted protein sequence before or within the DNA-binding WRC domain with little effort (in most cases, finding and verifying an appropriate allele required testing less than 30 plants). Nearly all guides we used caused high rates of mutations when expressed individually, even though they were selected from a large database without considering features such as sequence composition or secondary structure.

Investigating the molecular mechanisms of cellular processes or the function of gene families more often than not requires working with complex, multiple mutant genetic backgrounds. Multiplexed CRISPR-based screens promise a time-effective and flexible method for generating such backgrounds (reviewed in Najera et al., 2019; Hassan et al., 2021; Gaillochet et al., 2021). A handful of successful experiments of this kind have been reported with transgenic plants (Ma et al., 2015; Xie et al., 2015; Zhang et al., 2016); in a recent example, Suttmann et al. (2021) simultaneously expressed 24 sgRNAs in *Arabidopsis* and found that the rates of induced mutations were not uniform but rather varied substantially between different target genes. We observed the same phenomenon. Further, our data enabled us to roughly trace the trajectory of mutation rates at different GRF loci in plants expressing different numbers (one, two, four or eight) of sgRNA species. The results reveal that multiplexing was associated with pronounced disparities: while some targets consistently maintained high frequencies of induced mutations, mutation rates at other targets dropped, often drastically, with increasing number sgRNA species (Figure 7). Conceivably, this effect could be caused by synthetic lethality: assuming that certain multiple mutant genotypes arising in the mutagenesis are eliminated early in the life cycle, the overall rate and spectrum of mutations represented in the surviving seedlings would be skewed. Synthetic interactions are most commonly observed with closely related paralogs that provide overlapping or equivalent function; however, double mutants of closely related GRF sister genes (*grf1-3;grf2-10*, *grf3-9;grf4-17*, *grf5-3;grf6-8*, and *grf7-45;grf8-61*) were viable and fertile. Moreover, a survey of more than 1,000 individuals derived from plants that expressed sgRNAs targeting GRF1 through GRF8 and were also heterozygous for the *grf9-6* mutation (siblings of the seedlings analyzed by amplicon sequencing) uncovered only a single individual segregating for embryo-lethality and no individual displaying hallmarks of female gametophytic lethality (male gametophytes were not examined). Previously generated multiple mutant combinations of GRF insertion-alleles similarly show only relatively mild developmental defects (Kim et al., 2003; Horiguchi et al., 2005; Hewezi et al., 2012; Kim et al., 2012; Lee et al., 2012), such that there is little evidence to suggest pervasive synthetic-lethal interactions between GRF mutations.

It thus is more likely that uniform mutation rates were not achieved because of technical reasons. Many factors have an impact on the efficiency of genome editing, including the abundance of CRISPR/Cas9 complexes, the sequence composition of guide and PAM, the activity of DNA repair pathways, and the chromatin state at target loci (discussed in Sledzinski et al., 2021). In our experiment, mutation rates were relatively high when GRF genes were targeted individually, with only small differences between the sgRNA species we used (except for the guide targeting GRF8, which performed poorly throughout); but these small differences were vastly inflated upon multiplexing. What may have limited mutation rates at some loci over others? Xie et al. (2015) reported an analogous, if less pronounced interference effect when multiplexing sgRNAs in rice protoplasts and suggested competition of sgRNAs species for a limiting amount of Cas9 apoprotein as a possible cause. An alternative, though related explanation may be that different sgRNA species accumulate to different steady-state levels in the cell, for example, because they are more or less liable to degradation. Both scenarios would imply that different sgRNA species may not become represented equally in the pool of active CRISPR/Cas9 complexes, a possibility that could drastically influence editing frequencies.

Even if all sgRNA species are represented equally, multiplexing would be predicted to result in a dispersion effect: while the CRISPR/Cas9 complexes of plants expressing a single sgRNA species are uniform, and all Cas9 activity is directed against one target, plants expressing multiple sgRNA species assemble multiple different complexes, and only a fraction of the available Cas9 activity is directed against each specific target. This may enable other factors to play a more dominant role in determining editing efficiency. For example, CRISPR/Cas9 occupancy at eukaryotic target loci is dependent on the chromatin environment, and DNA-cleavage by Cas9 inhibited by the presence of nucleosomes (reviewed in Verkuijl and Rots, 2019). Weiss et al. (2022) recently reported a up to 200-fold lower frequency of editing events in regions of the *Arabidopsis* genome characterized by heterochromatic marks compared to regions with open chromatin. Liu et al. (2019) made similar observations in rice; moreover, they could show that adding a transcriptional activation domain to the Cas9 protein significantly improved editing frequencies, presumably through favorably impacting the chromatin state. A more or less open chromatin conformation may well have contributed to the relatively small differences in mutation rates we observed when targeting GRF genes individually and may have played a part in driving stronger disparities upon multiplexing.

It should be emphasized that these explanations, while perhaps plausible, remain largely untested. How (or even if) the variable 5′ targeting sequences may influence sgRNA processing, turn-over or association with the Cas9 apoprotein is not well understood (for a discussion see Jiang and Dounda, 2017; Allen et al., 2021). Similarly, it is difficult to estimate how factors such as the abundance of different CRISPR/Cas9 complexes, the sequence composition of guide and PAM, or the accessibility of target chromatin may interact to affect the frequency of editing events. Our experience with the GRF family would suggest that clarifying these details will benefit combinatorial screening or targeted mutagenesis strategies that rely on sgRNA multiplexing.

## Data Availability

TAnnotated sequence listings of the T-DNA vectors and helper plasmids created as part of this study are available in Supplementary Data Sheet S1, and plasmid DNA has been deposited with Adgene (addgene.com) as well as the Arabidopsis Stock Center, Ohio (ABRC, abrc.osu.edu): pCEE, 159746 & CD3-2850; pCUU, 159747 & CD3-2853; pTEE, 159748 & CD3-2852; pTUU, 159749 & CD3-2855; pYEE, 159750 & CD3-2851; pYUU, 159751 & CD3-2854; pGEM-2t: 159752 & CD3-2856; plasmids together with seed stocks that can be used as positive controls for fluorescence-based selection are also available as Education Kit CS27967. GRF reference alleles and double mutant lines can be obtained from ABCR: grf1-3, CS72426; grf2-10, CS72427; grf3-9, CS72428; grf4-17, CS72429; grf5-3, CS72430; grf6-9, CS72431; grf7-45, CS72432; grf8-61, CS72433; grf9-6, CS72434; grf1-3;grf2-10, grf1-3;grf2-10: CS73517; grf3-9;grf4-17, CS73518; grf5-3;grf6-8, CS73519; and grf7-45;8-61, CS73520; grf3-9;grf4-17, grf1-3;grf2-10: CS73517; grf3-9;grf4-17, CS73518; grf5-3;grf6-8, CS73519; and grf7-45;8-61, CS73520; grf5-3;grf6-8, grf1-3;grf2-10: CS73517; grf3-9;grf4-17, CS73518; grf5-3;grf6-8, CS73519; and grf7-45;8-61, CS73520; and grf7-45;grf8-61, grf1-3;grf2-10: CS73517; grf3-9;grf4-17, CS73518; grf5-3;grf6-8, CS73519; and grf7-45;8-61, CS73520.
