# Supplementary material for: CRISPR/Cas9 mutagenesis of the Arabidopsis GROWTH-REGULATING FACTOR (GRF) gene family
Source: Front Genome Ed. 2023 Oct 16;5:1251557. doi: 10.3389/fgeed.2023.1251557 (PMC10613670; doi:10.3389/fgeed.2023.1251557)
Supplement: Supplementary file 1 [file Table1.pdf]

Angulo et al., CRISPR/Cas9-induced mutagenesis of the Arabidopsis GRF family

SUPPLEMENTARY TABLE 1. Representation of *grf9* alleles in the amplicons

	Seedlings	Reads	Reads per copy[1]	
**'4-targets' sample**				
All	1151	66145	Expected:	29
grf9-3 & grf9-4	24	1411		29
grf9-1	12	567		47
grf9-7 & grf9-8	6	413		34
			Average:	37 ± 9
**'8-targets sample**				
All	1310	60496	Expected:	23
grf9-3 & grf9-4	24	1268		26
grf9-1	12	567		48
grf9-7 & grf9-8	6	413		39
			Average:	38 ± 11

[1] The number of reads generated by one allele of one seedling in the sample; the expected value is all mapped reads divided by two-times the number of seedlings in the sample. The observed values are the number of mutant reads divided by two-times the number of seedlings harboring the mutant alleles; an exception is *grf9-1*, which was calculated as number of mutant reads divided by the number of seedlings from *grf9-1*/*grf9-2* parents (since *grf9-2* cannot be amplified by our primers). Average ± standard deviation of the three observed values are indicated for both samples.

SUPPLEMENTARY TABLE 2. Summary statistics of amplicon sequencing

	GRF1	GRF2	GRF3	GRF4	GRF5	GRF6	GRF7	GRF8
**4sgRNA sample**								
Mapping rate	.97	.97	.98	.95	.97	.96	.97	.91
Mapped reads	114483	123969	99432	106500	114976	92153	84857	77923
Reads per copy[1]	50	54	43	46	50	40	37	34
Basis[2]	52662	57026	49716	53250	52889	42390	42429	38962
Indels[3]	1112	45027	1277	22327	3872	16865	0	5779
Mutation rate[4]	.02	.79	.03	.42	.07	.40	0	.15
	.02	*.83*	*.03*		*.07*	*.47*	*0*	*.12*
**8sgRNA sample**								
Mapping rate	0.95	0.94	0.97	0.93	0.97	0.94	0.97	0.96
Mapped reads	79337	120604	145741	128946	134287	59646	208216	133091
Reads per copy[1]	29	46	56	49	51	23	79	51
Basis[2]	76957	116986	141369	125078	130258	57857	201970	129098
Indels[3]	871	89789	330	35892	5801	18986	0	14059
Mutation rate[4]	.01	.77	<.01	.29	.04	.32	0	.11
	.01	*.85*	*.01*		*.05*	*.45*	*0*	*.16*

1 The number of reads expected to be generated by one allele of one seedling in the sample; calculated by dividing *mapped reads* by two-times the number of seedlings in the sample (2 x 1151 for '4sgRNA'; 2 x 1310 for '8sgRNA').

2 The number of reads expected from seedlings that had been mutagenized for a given GRF locus. In the '4sgRNA' sample, 527 seedlings (46%) were from parents harboring the '1256' construct, 582 (50%) from parents harboring the '3478' construct, and 42 (4%) were control seedlings; *basis* is calculated as *mapped reads x 0.46* for GRF1, GRF2, GRF5, GRF6, and *mapped reads x 0.5* for GRF3, GRF4, GRF7, GRF8. In the '8sgRNA' sample, 1265 seedlings (97%) were from parents harboring both constructs, and 45 (3%) were control seedlings; thus, *basis* is calculated as *mapped reads x 0.97*.

3 Insertion-deletion events detected by AGEseq; events supported by fewer reads than *reads per allele* were considered artifacts and not included.

4 Calculated as *indels / basis*. To validate the estimates, mutation frequencies were also estimated using the mapping and SNP-calling functions of Geneious 10.1.2 (https://www.geneious.com); the results are listed in cursive below the AGEseq estimates.
