# Supplementary material for: CRISPR/Cas9 mutagenesis of the Arabidopsis GROWTH-REGULATING FACTOR (GRF) gene family
Source: Front Genome Ed. 2023 Oct 16;5:1251557. doi: 10.3389/fgeed.2023.1251557 (PMC10613670; doi:10.3389/fgeed.2023.1251557)
Supplement: Supplementary file 5 [file Image1.PDF]

Angulo et al., CRISPR/Cas9-induced mutagenesis of the Arabidopsis GRF family

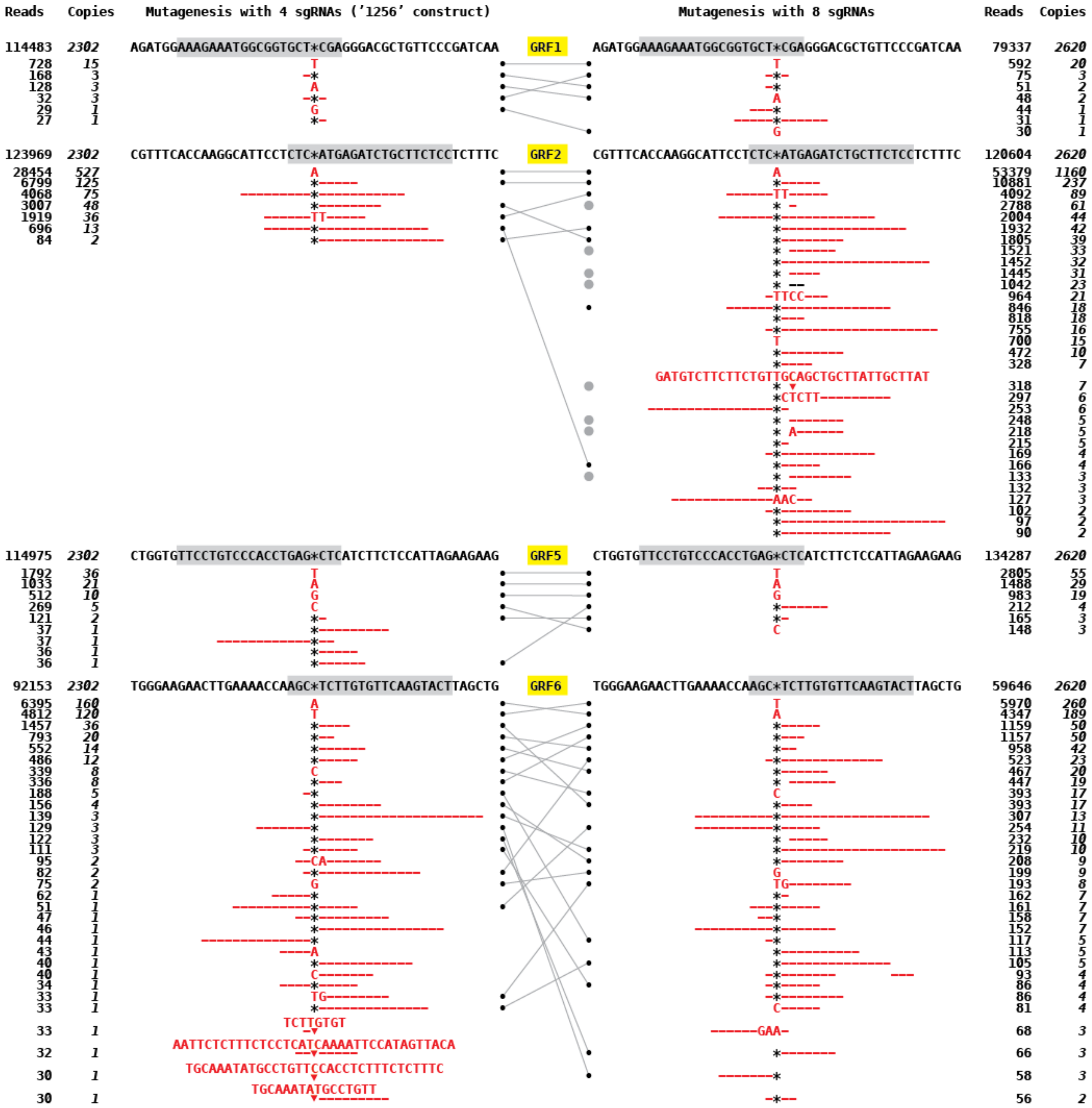

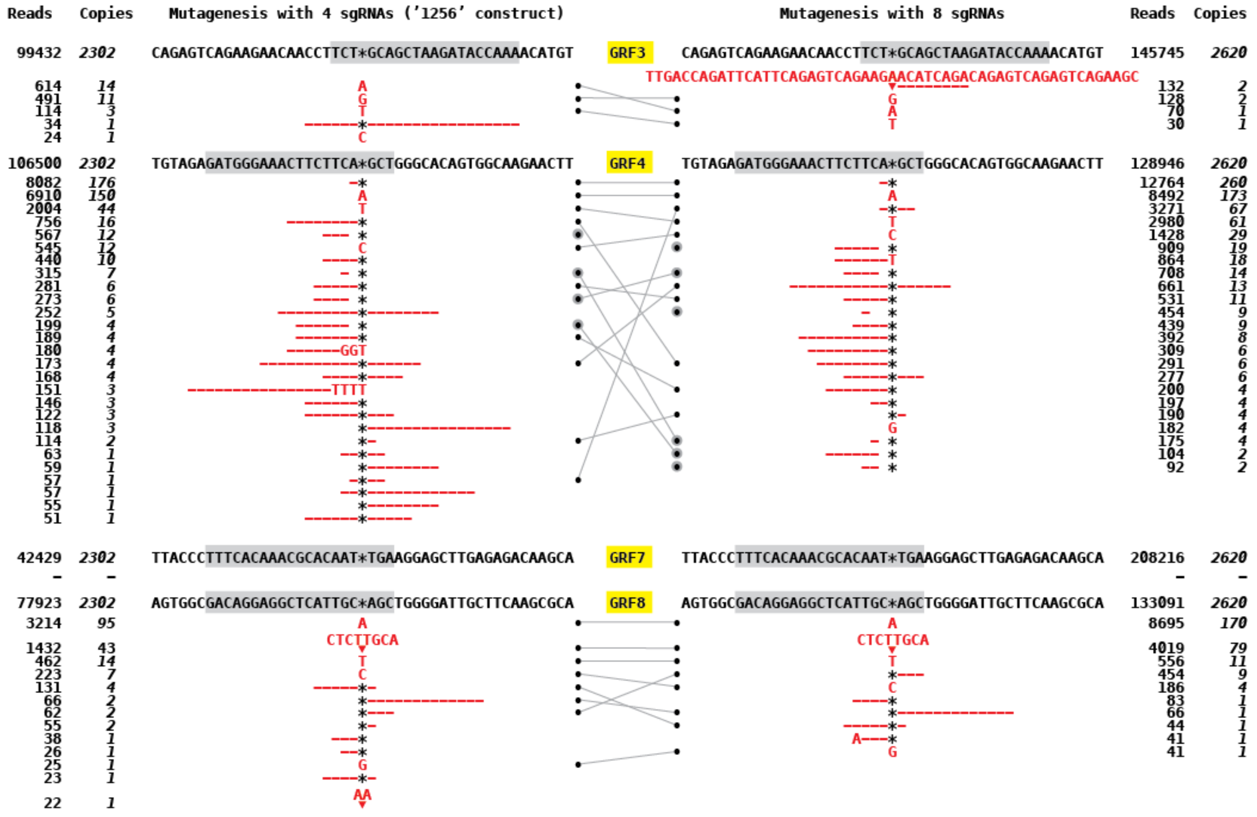

Supplementary Figure 1: Spectrum of CRISRP/Cas9-induced mutations at target genes. Spectrum and frequency of insertion-deletion events identified by AGEseq (Xue and Tsai, 2015) in GRF target genes (not that base pair transitions and transversions were not considered in the analysis, as they are not commonly induced by CRISPR/Cas9 and can be difficult to distinguish form PCR or sequencing artifacts; deletions of ~100 bp or greater would have escaped our assay). Targets are arranged according to their position on the '1256' or '3478' constructs, the protospacer regions are highlighted in grey, and the predicted Cas9 cut site is represented by a star. The total number of mapped reads supporting an allele is listed under 'reads', and the estimated number of seedlings in the pool that are heterozygous for the allele is shown in cursive under 'copies' (see Materials and Methods). Alleles that were found in both DNA pools are marked with a black dot and connected by grey lines; these alleles may not have been induced independently, since the same T1 plants gave rise to both pools. Grey dots mark alleles in which the predicted CRISPR/Cas9 cut site is not part of the lesion. The 35 bp insertion in GRF2 (GATGTC…) and the 16 bp insertions in GRF6 two alleles (TGAAAA…) originate from within the gene.
